# Robust behavioral assessment of the inducible Friedreich's ataxia mouse does not show improvement with NRF2 induction

**DOI:** 10.1242/dmm.052128

**Published:** 2025-04-02

**Authors:** Claire B. Montgomery, Lili Salinas, Garrett P. Cox, Lauren E. Adcock, Tiffany Chang, Francisco Figueroa, Gino Cortopassi, Elena N. Dedkova

**Affiliations:** ^1^ Department of Molecular Biosciences, University of California, Davis, Davis, CA 95616, USA; ^2^ Biomedical Research Models Inc., Richmond, CA 94806, USA; ^3^ Department of Basic Sciences, California Northstate University, Elk Grove, CA 95757, USA

**Keywords:** Friedreich's ataxia, Behavioral assessment, Muscular loss, NRF2 induction, Small-molecule therapeutics

## Abstract

Friedreich's ataxia, a recessive disorder caused by a mutation in the frataxin (*FXN*) gene, has few mouse models that demonstrate a progressive behavioral decline paralleling that of patients. A mouse model of systemic frataxin deficiency, the FXNKD, was recently developed using a doxycycline-inducible method; it is thought to mimic the patient phenotype seen when frataxin levels are decreased, but it has not been determined whether it is reliable for assessment of therapeutics. FXNKD mice underwent testing for 12 weeks alongside littermates, undergoing tests of motor function, gait and sensation. Additionally, a subset underwent treatment with omaveloxolone or dimethyl fumarate, both NRF2 inducers. We identified multiple techniques that sensitively detect decline in the mice, including open field, gait analysis and Von Frey tests. Furthermore, we developed a novel Salinas–Montgomery ataxia scale, which allows for more comprehensive assessment than a four-part cerebellar ataxia scale. Despite validating multiple sensitive techniques, we did not see any benefits of NRF2-inducing therapies in any tests. This was exacerbated by the discovery of a sexual dimorphism in FXNKD mice, in which males show more significant decline and better responsiveness to NRF2-inducing therapeutics.

## Teaser

**Summary:** Our mouse model of systemic frataxin deficiency shows a similar decline to patients with Friedreich's ataxia, with males showing faster decline in motor function compared to females, and no benefits from NRF2 induction with omaveloxelone or dimethyl fumarate.

## INTRODUCTION

Friedreich's ataxia (FA), an autosomal recessive disorder caused in almost all cases by a GAA triplicate expansion in intron 1 of the frataxin (*FXN*) gene, is characterized as a progressive cerebellar ataxia. This expansion is almost always homozygous, with both alleles expressing an average pathogenic GAA expansion of 703 bp on the shorter allele and 940 bp on the longer allele (La Pean et al., 2008). By contrast, unaffected individuals average 6-34 GAA repeats on each allele (Campuzano et al., 1996). FA is disease affecting 1 in 40,000 globally and 1 in 50,000 in the USA, with onset usually occurring in adolescence, between 10 and 15 years, but it can occur as late as in the 40s or 50s (Williams and De Jesus, 2023). It has been discovered that GAA expansion number influences the age of onset, with longer expansions causing earlier disease onset (Alper and Narayanan, 2003). Patients decline over the following years and require mobility assistance as their disease progresses. There are very few mouse models that truly parallel the physical decline of patients with FA, and, unfortunately, simple introduction of a murine GAA repeat series in mice does not cause any overt phenotype and does not cause the progressive decline or early-onset death seen in humans (Miranda et al., 2002). Additionally, genomic insertion of a humanized GAA expansion causes a very mild phenotype regardless of expansion length and does not result in the severe cardiomyopathy of the human disease (Bouchard et al., 2023).

FA was first reported in the 19th century, but, despite over a century of knowledge and research, mouse models with a correlated physical (Al-Madhawi et al., 2006), sensory (Piguet et al., 2018) and cardiac (Puccio et al., 2001) decline did not exist until the last 25 years owing to embryonic lethality or murine gene-editing incompatibilities, as frataxin is necessary during early development (Cossée et al., 2000). In previous years, multiple models have been created that develop an ataxic phenotype, either through short hairpin RNA (shRNA) insertions or humanized transgene insertions, that recapitulate multiple aspects of FA, but not the complete FA phenotype (Friedreich's Ataxia Research Alliance).

In this study, we characterized the progressive ataxic phenotype seen in the doxycycline (Doxy)-inducible shFXN mouse model (FXNKD) to define the degree of decline that can be achieved using a hands-off, chow-based approach. The FXNKD mouse has systemic knockdown of frataxin caused by genomic integration of a shRNA transgene under control of the H1 promoter, allowing transcription of these transgenes in the presence of Doxy (Chandran et al., 2017). Although the original study (Chandran et al., 2017) was completed using Doxy in drinking water and given by injection weekly, this required intraperitoneal (I.P.) injections with Doxy (5 mg/kg body weight) twice a week for 10 weeks followed by 10 mg Doxy/kg body weight twice a week for 2 weeks for comparable study animals. Excess handling, discomfort or adverse reactions to injections, and lack of palatability in water decreasing overall water intake can cause many sources of variability as well as unnecessary discomfort to the mice. It has been found that induction can be successful using *ad libitum* chow feeding of 200 ppm Doxy, preventing the need for weekly injections and unnecessary animal handling (Mercado-Ayón et al., 2022; Perry et al., 2024).

The existing studies in the FXNKD mouse (referred to here as ‘TG^+^’) only quantified behavior in a few tests (rotarod, grip strength and manual gait analysis). This study sought to characterize both the loss of muscular dexterity and decline in peripheral sensation to better understand the phenotype of the TG^+^ mouse and its correlation with the human phenotype (Chandran et al., 2017; Mercado-Ayón et al., 2022; Perry et al., 2024). Additionally, this study sought to assess the capacity for therapeutic testing in the FXNKD mouse. At the time of writing, treatment with omaveloxolone, an NRF2 (also known as NFE2L2)-inducing drug, is the only on-market treatment for patients with FA.

Activation of the NRF2 pathway by translocation of NRF2 from KEAP1 has been shown to decrease in FA as a result of increased ferroptosis and lipid peroxidation seen in FA cells (Sanz-Alcázar et al., 2024). Translocation of NRF2 to the nucleus causes activation of the antioxidant response element and transcription of multiple signaling molecules downstream, including NAD(P)H dehydrogenase quinone 1 (*NQO1*), glutathione (GSH) and heme oxygenase 1 (*HMOX1*) (Lin et al., 2023).

This study tested the capacity for therapeutic drug testing in the TG^+^ mouse by evaluating the effects of two NRF2-inducing drugs – omaveloxolone (OMAV) and dimethyl fumarate (DMF) – on phenotypic decline (Lee, 2023). Treatment with these molecules has been shown to increase NRF2 signaling pathway induction and activation of the antioxidative stress response, and is hypothesized to improve the behavioral decline seen in FA. Because patients in the OMAV clinical trial showed a modest rescue in clinical FA scoring outcomes short term (12 weeks; Lynch et al., 2018), we assessed animals at weekly or regular intervals to assess the phenotypic decline of animals with or without NRF2 induction.

We confirmed induction of NRF2 with the treatments after completing behavior testing by mRNA transcript levels of downstream markers in males, which were rescued to wild-type (WT) littermate levels; however, no significant improvement in motor or sensory deficits was observed with either drug. Additionally, female TG^+^ mice did not respond to NRF2-inducing therapies, nor did they show a significant difference in baseline downstream signaling levels, suggesting a sexual dimorphism in NRF2 response and signaling. In discovering this dimorphism at an earlier timepoint than previously published (Perry et al., 2024), we then analyzed the sexes separately to assess the effects of these therapies on behavioral outcomes. Assessment of the sexes separately also revealed a trend toward differential phenotype in males and females, previously only seen at 18 weeks of induction, as well as significantly different survival outcomes, likely driven by differences in knockdown of frataxin in the cerebellum (Perry et al., 2024).

## RESULTS

### TG^+^ mice display significant knockdown of *Fxn* and physical signs of wasting

**Fig. 1.**
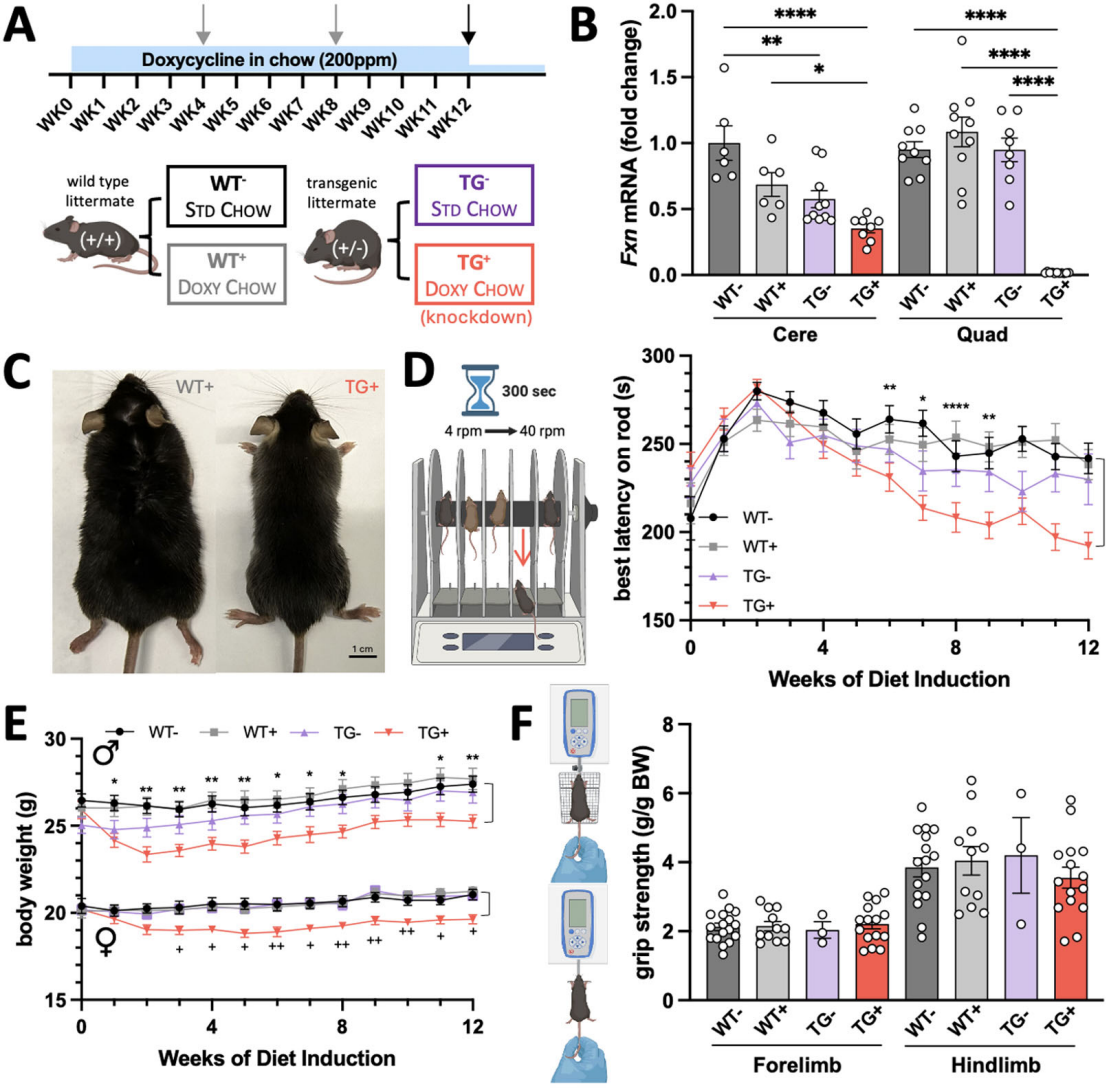
**Confirming deficits in FXNKD (TG^+^) mice with doxycycline (Doxy) administration in chow.** (A) Top: timeline for Doxy administration of mice, in which 12- to 15-week-old mice were treated with Doxy starting at week 0 of study, and behavior was tested at the points indicated by gray/black arrows. Mice used for biochemical analysis were euthanized at the black arrow, and survival analysis animals continued on Doxy until humane endpoint. Bottom: groups evaluated by genotype and chow type, and the nomenclature used to describe each. STD, standard; WK, week; WT, wild type. Created in BioRender by Montgomery, C. B. (2024). https://BioRender.com/958wd89. This figure was sublicensed under CC-BY 4.0 terms. (B) Confirmation of *Fxn* knockdown by quantitative real-time PCR in TG^+^ mice after 12 weeks of Doxy treatment in two target tissues: cerebellum and quadricep muscle (*n*=6-11 per bar). (C) An image of a WT^+^ male next to a TG^+^ male after 12 weeks of Doxy administration, to scale. (D) Rotarod test results, with schematic shown on the left, over 12 weeks of Doxy administration. Significance refers to WT^−^ versus TG^+^ (*n*=21-45 per curve). Created in BioRender by Montgomery, C. B. (2024). https://BioRender.com/a54c180. This figure was sublicensed under CC-BY 4.0 terms. (E) Body weight comparison of all four groups across both sexes (males, top; females, bottom). ‘*’, WT^−^ male versus TG^+^ male at given timepoint; ‘^+^’, WT^−^ female versus TG^+^ female at given timepoint (*n*=8-16 per curve). (F) Normalized grip strength testing was completed as described after 12 weeks of Doxy induction (*n*=3-17 per bar). BW, body weight. Created in BioRender by Montgomery, C. B. (2024). https://BioRender.com/a54c180. This figure was sublicensed under CC-BY 4.0 terms. B,D, one-way ANOVA; E, mixed effects two-way ANOVA; F, two-way ANOVA, followed by Tukey's multiple comparisons tests (*P<0.05, **P<0.01, ****P<0.0001; ^+^*P*<0.05, *^++^P*<0.01).

The physical performance and appearance of TG**^+^** mice (FXNKD) were compared after 12 weeks of Doxy induction with those of three ‘phenotypically normal’ littermate controls: WT**^−^** (no transgene, no Doxy), WT**^+^** (no transgene, with Doxy) and TG**^−^** (transgene present, no Doxy). These groups are described in Fig. 1A and Table 1. First, we compared our findings with those of the original study by Chandran et al. (2017), which was completed with combined water dosing/I.P. injection with induction through *ad libitum* chow-based medium. As shown in Fig. 1B, induction through Doxy chow still creates significant knockdown of the *Fxn* gene, with only 35% of WT**^−^** level remaining in cerebellar tissue by the 12th week of induction. Although FA is characterized as a cerebellar ataxia, we observed the greatest knockdown of *Fxn* expression in the quadricep muscle (1.2% of WT**^−^** *Fxn* level). Compared to all three similar, healthy controls, the TG**^+^** mouse appeared more frail, thin and bony, and showed signs of accelerated aging, such as graying of the coat (Fig. 1C).

**Table 1.** Descriptions of the nomenclature used, and the genotypes/treatments associated with each group identifier

Nomenclature	Description of genotype/treatment
WT^−^	Wild-type littermate, no doxycycline treatment
WT^+^	Wild-type littermate, treated with doxycycline
TG^−^	Transgenic animal (one copy of shRNA), no doxycycline treatment
TG^+^	Transgenic animal (one copy of shRNA), treated with doxycycline (‘knockdown’)

### TG^+^ phenotype is not the same when induced in chow versus water and I.P. injection

We observed a similar, linear deficit in the rotarod endurance assay in TG**^+^** versus WT**^−^** mice as in other literature (Chandran et al., 2017; Mercado-Ayón et al., 2022; Perry et al., 2024), showing a significant deficit by 6 weeks of Doxy induction (Fig. 1D). However, TG**^−^** animals also appeared to be slightly impaired compared to WT**^−^** and WT**^+^** littermates, although not significantly, like the impairments shown in *Fxn* gene expression (Fig. 1B) and body weight (Fig. 1E) compared to those of WT**^−^** and WT**^+^** littermates.

We hypothesized that water and chow induction would have a similar outcome, but, compared to the original article (Chandran et al., 2017), we did not see deficit in any parameters of grip strength. Against our hypothesis, all methods of normalized grip strength testing (four limbs, front limbs only or hindlimbs only) showed insignificant differences across all genotypes (Fig. 1F; Table S1). There was not a significant main effect of genotype/treatment group on grip strength measurements (*P*=0.6413; Table S2). Because this test is normalized to body weight, a difference in grip strength that is proportionally consistent with body weight difference will negate the potential deficits and therefore is not a good identifier of strength loss in the FXNKD mouse model. Unnormalized data are also included in Fig. S1, which have been reported in other models of FA to show a trend in impairment (Fil et al., 2023), but did not show a significant difference between groups for forelimbs or hindlimbs.

### Additional measures of gait and limb dysfunction in the FXNKD mouse

**Fig. 2.**
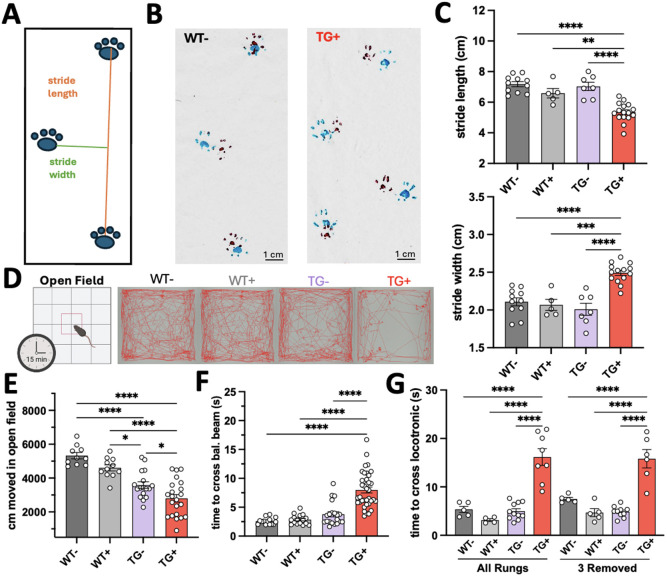
**Gait and limb coordination testing in the FXNKD mouse model after 12 weeks of Doxy administration.** (A) Demonstrative gait trace with measurement identifiers showing stride length and stride width (perpendicular to length). (B) Representative gait traces from a WT^−^ and TG^+^ mouse (front paws painted red, hind paws painted blue). (C) Stride length (top) and stride width (bottom) averaged from measurements between both forepaws and both hindpaws of a subset of tested mice (*n*=5-16 per bar). (D) Representative traces from the open field apparatus, recorded from overhead and automatically produced by the analysis software, show the total ambulation pattern over 15 min from a single mouse of each genotype. Created in BioRender by Montgomery, C. B. (2024). https://BioRender.com/gn8i987. This figure was sublicensed under CC-BY 4.0 terms. (E) Open field total distance traveled in 15 min of testing (*n*=12-27 per bar). (F) Time to cross a 100 cm length of 16 mm diameter beam was reported (*n*=19-30 per bar). (G) Time to cross the Locotronic foot misplacement apparatus was tested in a subset of mice (*n*=4-11 per bar). C,E,F, one-way ANOVA followed with Tukey's multiple comparisons tests; G, two-way ANOVA followed by Tukey's multiple comparisons tests (*P<0.05, **P<0.01, ***P<0.001, ****P<0.0001).

As also shown in the original FXNKD paper (Chandran et al., 2017), we assessed gait by the ‘manual’ method (Fig. 2A). Animals had both front paws (red) and hind paws (blue) painted, and were allowed to walk across exam paper, multiple times if needed. As shown in the example traces (Fig. 2B), we were able to obtain multiple clear prints per animal to analyze. Within our analysis, we discovered that multiple measures of gait changed, not only stride length as shown previously (Chandran et al., 2017). Stride width showed a significantly bigger spread in the limbs of FXNKD animals (an average of outcomes between both front paws and both back paws), as we hypothesized, whereas average stride length showed shortening (Fig. 2C).

The open field test was completed with a differing apparatus setup than in the original paper by Chandran et al. (2017) (Fig. 2D). However, we saw a similar outcome, in that the TG**^+^** group showed a significant decrease in ambulation (Fig. 2E). Similar outcomes were seen in the balance beam 16 mm test (Fig. 2F) and Locotronic foot misplacement test (Fig. 2G), in which TG**^+^** mice took significantly longer than WT^−^, WT^+^ and TG^−^ mice to cross the designated zone. The Locotronic test showed a high level of variability, and there was not a significant main effect of genotype/treatment on time to cross the apparatus (*P*=0.6152; Table S2). The balance beam test showed a ‘plateau’ in potential maximum time to cross prior to 12 weeks of induction, likely due to the limit of beam length (Fig. S2A). Schematics of the balance beam and Locotronic testing apparatuses are also included in Fig. S2B.

### Assessment of peripheral nervous system and stability deficits

**Fig. 3.**
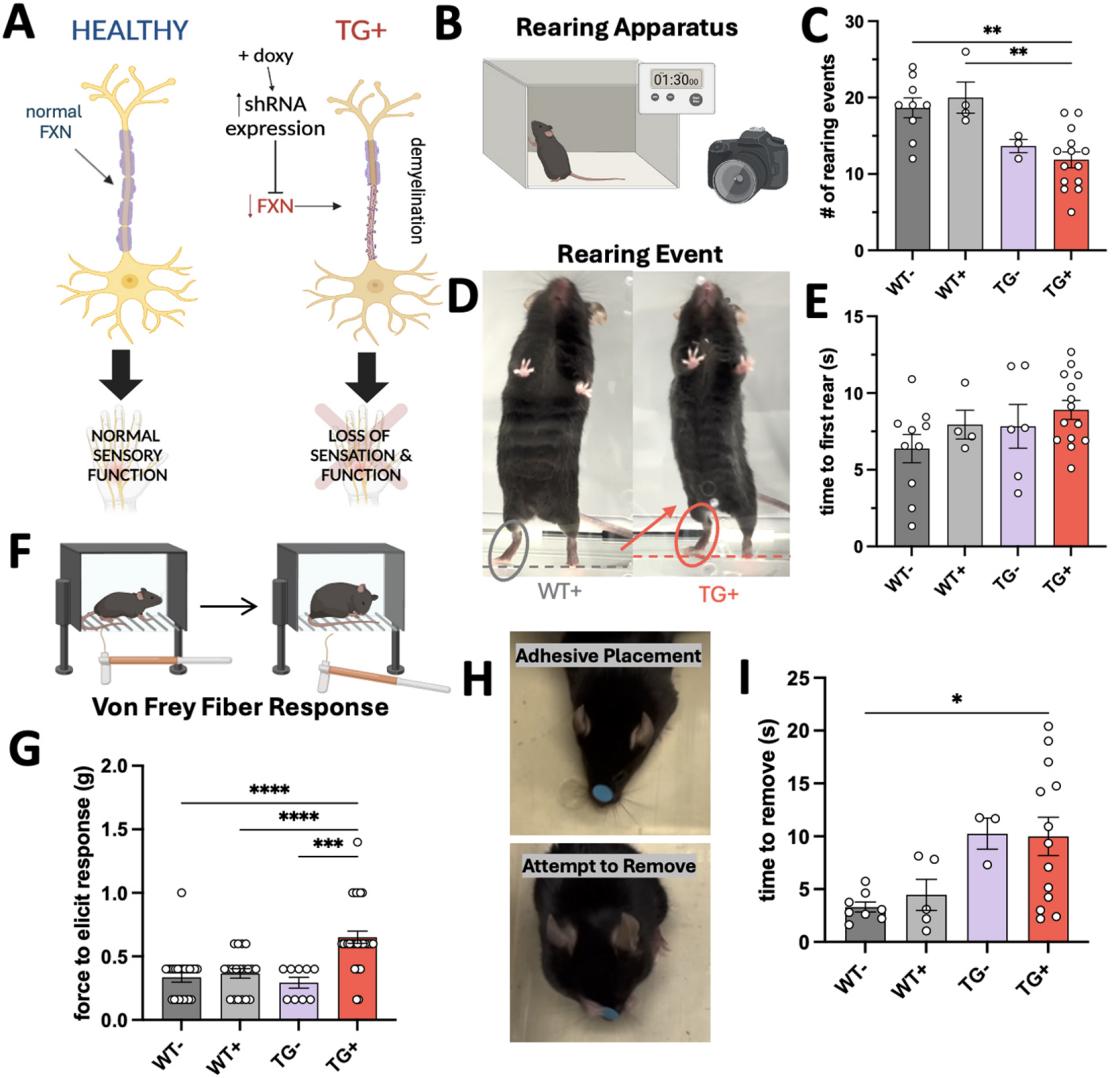
**Discovery of loss peripheral nerve sensation and upright stability in TG^+^ mice after 12 weeks of Doxy.** (A) Schematic describing the mechanism of sensory loss. Demyelination of sensory nerves and development of nerve lesions due to FXN protein deficiency causes a progressive decrease in peripheral sensation. Created in BioRender by Montgomery, C. B. (2024). https://BioRender.com/m77g760. This figure was sublicensed under CC-BY 4.0 terms. (B,D) Apparatus setup for the rearing test, in which one wall was clear, with a camera facing that wall, and examples of WT^+^ and TG^+^ mice displaying a rearing event. Created in BioRender by Montgomery, C. B. (2024). https://BioRender.com/x97j554. This figure was sublicensed under CC-BY 4.0 terms. (C,E) The number of rearing events in a 90 s testing period was assessed (C), as well the time needed for an animal to rear once placed in the chamber (E) (*n*=4-14 per bar). (F) Von Frey fiber testing was carried out using a manual Von Frey apparatus. Created in BioRender by Montgomery, C. B. (2024). https://BioRender.com/x97j554. This figure was sublicensed under CC-BY 4.0 terms. (G) Von Frey fiber testing was assessed by reporting the minimum force needed from five total trials per animal – four additional ‘up-down’ trials after first response (*n*=9-30 per bar). (H) Adhesive testing was completed using a sticker on the snout. Representative images of the placement (top) and attempted removal (bottom) of the adhesive. (I) Time to completely remove the adhesive stimulus from the snout was reported (*n*=3-13 per bar). C,E,G,I, one-way ANOVA followed by Tukey's multiple comparisons tests (*P<0.05, **P<0.01, ***P<0.001, ****P<0.0001).

The presence of stance changes in hindlimb gait analysis also created concern that peripheral sensation and balance/stability might be affected in these mice, paralleling the human condition. We sought to determine whether any measures of peripheral sensory function were affected, as peripheral sensation is significantly affected in humans, and many patients with FA present with symptoms of peripheral neuropathy (Indelicato et al., 2018). Sensory deficit in patients with FA is greatly associated with demyelination of neurons (Fig. 3A), which is thought to directly affect the prevalence of peripheral loss of innervation seen in most patients with FA (Creigh et al., 2019).

We completed a non-stimulated rearing test to assess peripheral stability (Fig. 3B), in which TG**^+^** animals showed a difference in exploration compared to their WT^−^ and WT**^+^** littermates after being placed in the chamber, represented by a significant decrease in total rearing events (Fig. 3C). Interestingly, TG**^+^** mice consistently appeared to stand further from the wall during rearing activities, which has not been previously studied, but suggests that these impaired animals need to stand at a further distance to improve balance during a rearing event, shown by the representative images in Fig. 3D. TG**^+^** mice also showed a trend toward increased time to initiate the first rear after being placed in the apparatus (Fig. 3E), but there was not a statistically significant main effect of genotype/treatment on rearing initiation time (*P*=0.1877; Table S2).

As confirmation of hindlimb sensory deficit, we completed Von Frey filament testing (Fig. 3F) using the up-down method on a hind paw (McMackin et al., 2016). Animals were given four more directed stimuli after the first response, and the minimum force needed to respond was reported (Fig. 3G). TG**^+^** mice displayed a significant sensory deficit at 12 weeks of Doxy induction, in which it took almost double the force to elicit a response from these mice compared to WT^−^, WT^+^ and TG^−^ mice.

We tested the phenotypic effects on the adhesive test, which is not used extensively in ataxia research but rather in models of hemiparesis or other single-limb injury to assess sensory loss (Schallert et al., 1982). Because we did not expect a limb-to-limb difference based on performance in other tests, we chose to test a single simulation point (snout) for each animal (Fig. 3H). As we hypothesized, the TG^+^ animals showed statistically significant impairment in the amount of time needed to complete the full removal of the adhesive (Fig. 3I). This result is consistent with the significant deficit observed in Von Frey sensation (Fig. 3G) and can also be completed multiple times over a study.

### Assessing qualitative, observational measurements of ataxia

To date, multiple observational measurements exist in clinical settings for patients with FA, whereas few exist in mice to study cerebellar ataxias. In human patients, qualitative scores are assigned by clinical observation of the patient completing a list of tasks, such as the Friedreich's ataxia rating scale (FARS) or scale for the assessment and rating of ataxia (SARA) (Bürk et al., 2013). Some of the most common qualitative scoring assays in mice are the frailty assessment (in aging animals) and the four-part cerebellar ataxia rating scale (Whitehead et al., 2014; Guyenet et al., 2010).

**Fig. 4.**
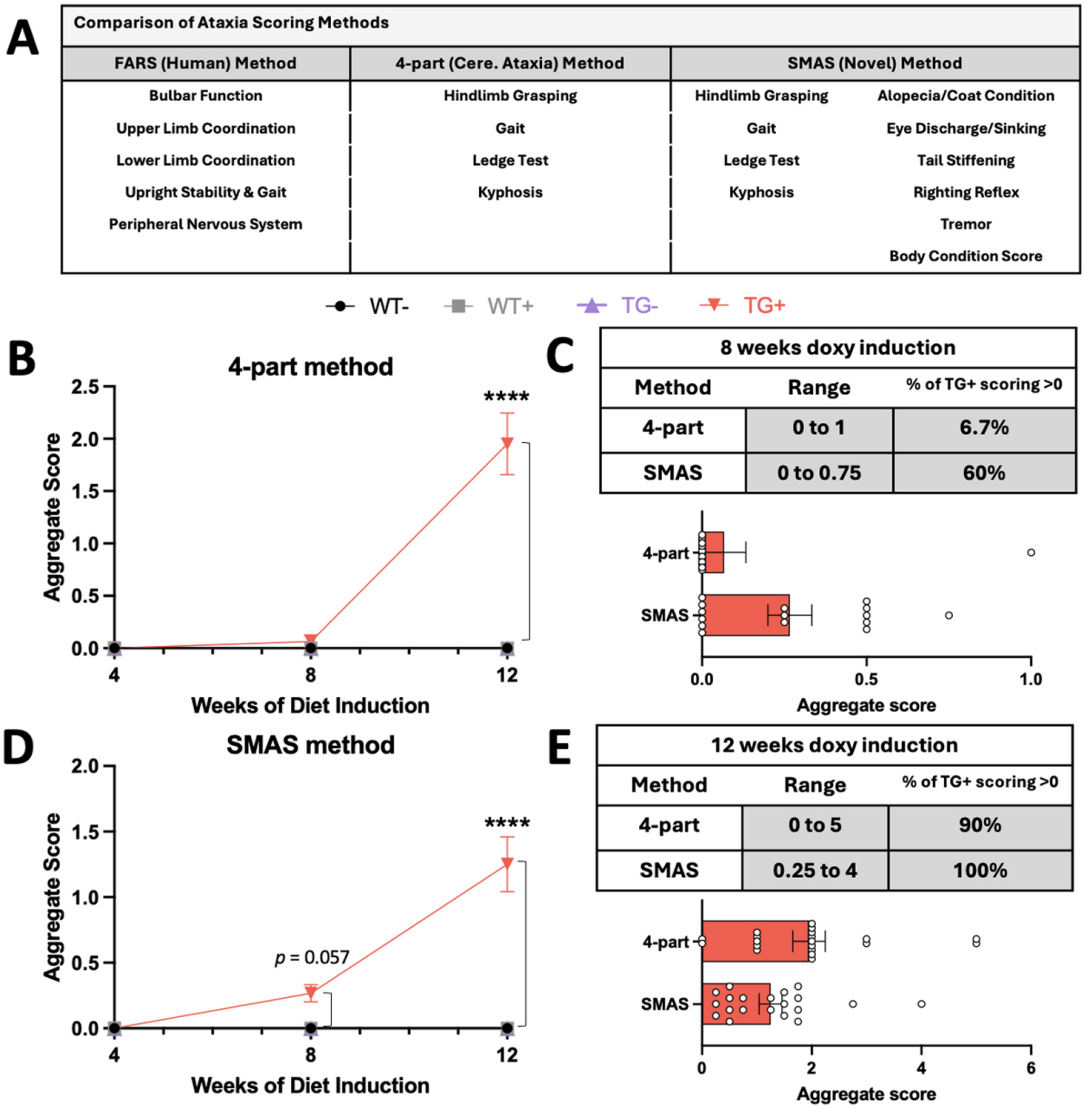
**Developing a less variable ataxia scoring method in Friedreich's ataxia (FA).** (A) Table comparing the readouts from human scoring [Friedreich's ataxia rating scale (FARS)], the classical mouse ataxia scoring (four-part method) in mice and the novel method developed and tested in-house [Salinas–Montgomery ataxia scale (SMAS)]. (B,D) A comparison of scoring outcomes using the four-part method (B) and SMAS method (D) in the same mice after 8 and 12 weeks of Doxy induction (mice do not show a visible phenotype at week 4). Significance markings refer to WT^−^ versus TG^+^ (*n*=4-15 per curve). (C,E) Assessment of overall ranges and box and whisker plots for the four-part method and SMAS method from TG^+^ subjects after 8 (C) and 12 (E) weeks of Doxy induction (*n*=15 per row/bar). All control group animals (WT^−^, WT^+^, TG^−^) had scores of zero at the timepoints tested. B,D, Kruskal–Wallis non-parametric test with Dunn's multiple comparisons test; C,E, plots display mean±s.e.m. (****P<0.0001).

As the TG**^+^** phenotype continued to progress, we noted that animals began to display signs that paralleled those seen in aging. Changes in coat condition and color (graying), changes to eye structure and discharge, and peripheral issues, such as tail strength and abnormal balance, were noted in multiple animals, so we sought to create a broader assay to non-invasively assess the progression of phenotype in the FXNKD mice. In doing so, we tried to mirror the FARS, while including the four-part cerebellar ataxia assessments, and implemented some measures seen in the frailty test (Fig. 4A). The frailty test's scoring breakdown was used as a model to score each parameter, in which more than three scoring outcomes are possible (Table 2). The four-part test utilizes integers only, from 0 to 3, to score severity of disease. By contrast, our test, which was developed by authors L.S. and C.B.M., adds six additional observations and utilizes subpoints the four-part test does not include, with potential scores from 0 to 1, shown by the ranges in Table 2, allowing for finer scoring and earlier identification of phenotype, and more potential scoring outcomes as animals continue in ataxia progression. Added points of analysis include assessment of coat condition, eye discharge or sinking, tail stiffening (measured by running a finger along the mouse tail and assessing stiffening/curling response), righting reflex (the ability of the mouse to right itself and land on all four paws when falling about 6 inches, dropped upside down), presence of tremor and body condition score (BCS; an indicator of loss of mass). A full overview of scoring details can be found in Table S3.

**Table 2.** Comparison of four-part and SMAS scoring

Four-part method	Unaffected	Mild	Moderate	Severe
Hindlimb grasping	0	1	2	3
Gait	0	1	2	3
Ledge test	0	1	2	3
Kyphosis	0	1	2	3
SMAS method	Unaffected	Mild	Moderate	Severe
Hindlimb grasping	0	0.25-0.5	1	1
Gait	0	0.25	0.5	1
Ledge test	0	0.25	0.5-1	1
Kyphosis	0	0.25	0.5	1
Alopecia/coat condition	0	0.5	0.5-1	1
Eye discharge/sinking	0	0.5	0.5	1
Tail stiffening	0	0.5	1	1
Righting reflex	0	0.5	0.5	1
Tremor	0	0.5	0.5	1
BCS	0	0	0.5	1

Possible scoring outcomes from four-part and SMAS tests when compared head-to-head. Because the SMAS test has additional intervals defining disease progression states, variability and dynamic range are much smaller. Additionally, there are more possible scoring outcomes in the mildly to moderately affected phase, which allows for earlier screening of deficit. Any listed ranges for scoring are further defined in Table S3. BCS, body condition score; SMAS, Salinas–Montgomery ataxia scale.

The four-part scale and Salinas–Montgomery ataxia scale (SMAS) had outcomes compared at 8 weeks of induction and 12 weeks of induction (Fig. 4B,D). At both timepoints, the SMAS showed a significant main effect of genotype/treatment on scoring outcome (8 weeks, *P*=0.01; 12 weeks *P*<0.0001), while the four-part method showed no significant main effect of genotype/treatment on scoring outcome at 8 weeks of induction (*P*>0.8315) but a significant main effect after 12 weeks (*P*<0.0001; Table S2). As shown in Fig. 4C,E, variability and range within the TG^+^ group was remarkedly decreased at both 8 and 12 weeks of Doxy with the SMAS test, compared with four-part outcomes from the same animals.

### Assessing NRF2 activation by utilizing newly optimized testing protocols

**Fig. 5.**
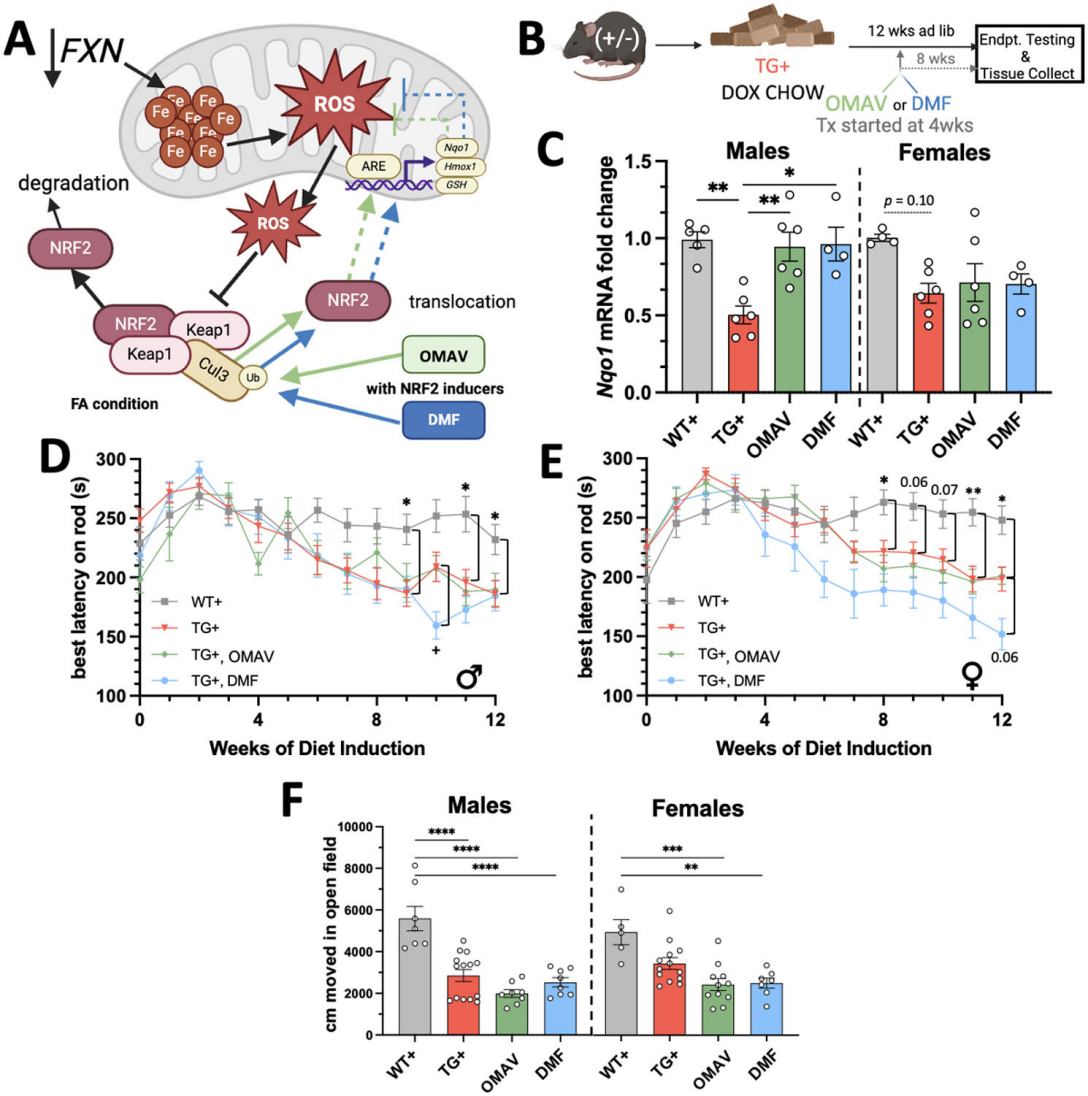
**NRF2-inducing drugs omaveloxolone (OMAV) and dimethyl fumarate (DMF) show no effect on TG^+^ phenotype.** (A) Schematic describing the hypothesized benefit of NRF2 induction in FA (black arrows indicate FA condition, blue/green arrows indicate hypothesized benefit). Created in BioRender by Montgomery, C. B. (2024). https://BioRender.com/k43s978. This figure was sublicensed under CC-BY 4.0 terms. (B) Schematic showing the induction and dosing paradigm of dosed mice, who were tested concurrently with mice that did not receive NRF2-inducing drugs and instead received oral vehicle dosing. Created in BioRender by Montgomery, C. B. (2024). https://BioRender.com/k43s978. This figure was sublicensed under CC-BY 4.0 terms. (C) mRNA expression in quadricep muscle of NRF2-downstream *Nqo1* (*n*=4-6 per bar). (D,E) Rotarod over 12 weeks of Doxy in males (D) and females (E), with OMAV/DMF treatment starting at week 4 of Doxy. ‘*’, WT^+^ versus TG^+^. OMAV treatment showed no significance at any timepoint versus TG^+^ group (*n*=8-23 per curve). (F) Open field test after 12 weeks of Doxy, 8 weeks of OMAV/DMF treatment (*n*=5-14 per bar). C,F,G, two-way ANOVA followed by Tukey's multiple comparisons tests; D,E, mixed effects two-way ANOVA followed by Tukey's multiple comparisons tests. WT^+^ and TG^+^ (no NRF2-inducing drug) animals overlap with Figs 1 and 2 in C-G but are split by sex (*P<0.05, **P<0.01, ***P<0.001, ****P<0.0001).

As we assessed the overall phenotype of the FXNKD mouse, we also sought to determine whether this mouse could be used to adequately assess treatment benefit on muscular outcomes. Induction of the NRF2 signaling pathway has been identified as a possible therapeutic mechanism in FA, which is proposed to decrease reactive oxygen species (ROS) and increase mitochondrial gene expression (Fig. 5A). We allowed TG**^+^** mice to begin the progressive decline for 4 weeks on Doxy chow alone, then provided an oral dose of NRF2 inducers daily. We assessed two NRF2 inducers: OMAV (SKYCLARYS^®^), the only on-market therapeutic for FA at the time of writing (Lee, 2023), and DMF (Hayashi et al., 2017). Both drugs were converted from their human therapeutic doses (150 mg OMAV and 480 mg DMF daily) to equivalent mouse dosages (24 mg kg^−1^ OMAV and 82 mg kg^−1^ DMF daily) using body surface area conversions (Reagan-Shaw et al., 2008). Drugs were administered once daily in afternoons to allow for a head-to-head comparison (Fig. 5B).

We quantified mRNA levels of an NRF2 downstream gene, *Nqo1*, in quadricep muscle, a main target tissue of functional loss. Males showed significant downregulation of *Nqo1* expression, and both OMAV and DMF significantly rescued the decreased *Nqo1* levels (Fig. 5C). By contrast, in females, *Nqo1* expression was not significantly downregulated, and both OMAV/DMF did not cause a change in *Nqo1* expression. Because such a stark difference in response was seen at the biochemical level, we sought to define the therapeutic effect of NRF2-inducing molecules on both sexes separately.

**Fig. 6.**
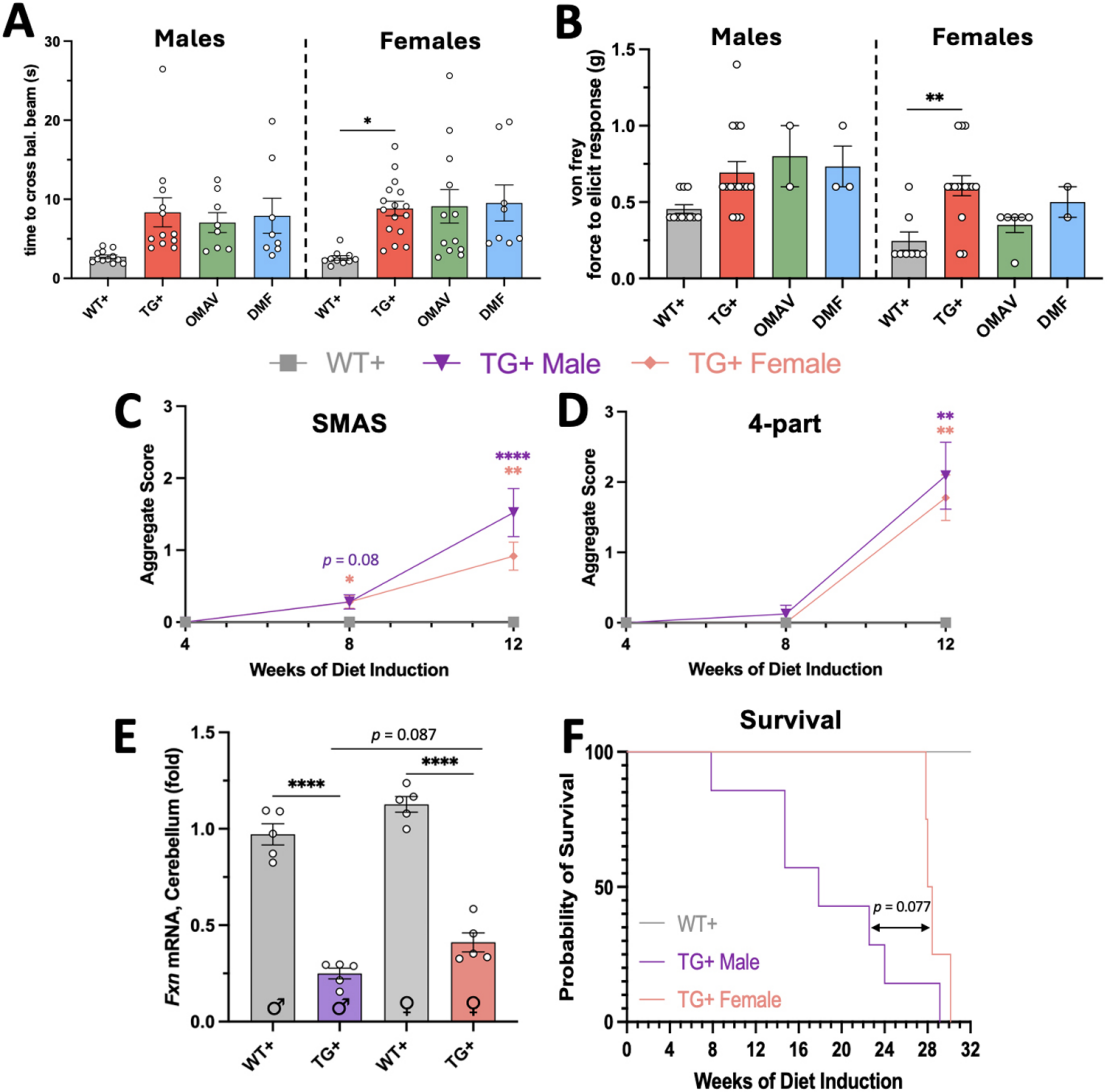
**NRF2 inducers have no effect on behavior, and sexual dimorphism is shown by mRNA levels and survival.** (A) The time to cross a 16 mm balance beam was reported after 12 weeks of Doxy, 8 weeks of drug treatment (*n*=8-16 per bar). (B) Von Frey force measurements after 12 weeks of Doxy, run on a subset of mice (*n*=2-15). (C,D) SMAS (C) and four-part testing (D) scores after 8 and 12 weeks of Doxy, with OMAV/DMF starting at week 4 (*n*=8-11 per point). (E) *Fxn* mRNA levels in homogenized cerebellar tissue after 12 weeks of Doxy, with OMAV/DMF starting at week 4 (*n*=5 per bar). (F) Survival of male and female TG^+^ mice through 32 weeks of Doxy (WT^+^, *n*=6; TG^+^ male, *n*=7; TG^+^ female, *n*=4). A,B,E, two-way ANOVA followed by Tukey's multiple comparisons tests; C,D, Kruskal–Wallis non-parametric test with Dunn's multiple comparisons test; F, Mantel-Cox test for survival. WT^+^ and TG^+^ (no NRF2-inducing drug) animals overlap with Figs 1-4 in A-F but are split by sex. (*P<0.05, **P<0.01, ****P<0.0001).

The potential therapeutic effect of NRF2 induction on muscular decline was assessed in parallel with all genotype studies in Figs 1-4, and control group values overlap with Figs 1-4 in Figs 5 and 6. Muscular endurance was not improved by either treatment in either sex, as shown by the progressive decline in latency in the rotarod assay, which did not indicate an improvement in performance over that of the non-dosed TG**^+^** mice (Fig. 5D). In fact, DMF appeared to cause an impairment in female mice, whereas OMAV caused no change (Fig. 5E).

Ambulatory ability was also assessed by the open field test after 12 weeks of induction, which showed no effect of either treatment on 15 min of ambulation (Fig. 5F). Both OMAV- and DMF-treated mice performed slightly worse in the open field than did the untreated mice. As confirmed, after 12 weeks of Doxy and 8 weeks of OMAV/DMF treatment, balance beam (Fig. 6A) and Von Frey (Fig. 6B) tests did not indicate that NRF2 induction rescues behavioral deficits in this mouse model.

### Evaluating the degree of sexual dimorphism observed after 12 weeks of Doxy induction

Because females showed a trend toward improved scores over males in rotarod, open field, balance beam and Von Frey tests, we compared observational testing scores using both scoring methods (Fig. 6C,D). TG^+^ females showed a trending decrease in aggregate ataxia scoring by SMAS compared with that of TG^+^ males, with no trend in four-part testing.

To identify why males may show a more accelerated disease profile, we quantified *Fxn* mRNA levels in multiple target tissues, with cerebellum showing a significant sex-dependent difference in expression levels (Fig. 6E). The quadricep muscle showed 1-2% residual *Fxn* expression remaining in both sexes (Fig. S3). Although the main effect of the interaction between sex and genotype was insignificant (*P*=0.9471), we found that the main effect of sex on cerebellar *Fxn* levels was significant (*P*=0.0026; Table S2). We hypothesized that the cerebellar decline could account for the acceleration of phenotype, and assessment of survival in a separate cohort of animals showed that male survival was significantly impaired compared to that of female counterparts, with males living a median of 17.2 weeks on Doxy (Fig. 6F). By contrast, no females died during the 24 weeks of study.

## DISCUSSION

FA is a progressive neurodegenerative disorder caused by downregulation of *FXN* transcript and resulting protein. FA is estimated to affect ∼1:50,000 people, with a carrier prevalence of ∼1:110, although recent molecular data have suggested a higher prevalence (Delatycki et al., 2000). Multiple mouse models of FA exist, with varying degrees of phenotypic decline, cardiac damage and early mortality. Approaches to creation of FA mouse models include Cre-Lox systems, human transgene insertions and Doxy-inducible systems, such as the FXNKD (TG^+^) mouse (Friedreich's Ataxia Research Alliance).

Full knockout of frataxin has been shown to cause embryonic lethality, and insertion of a diseased human transgene does not cause a robust phenotype mirroring that of human patients (Kalef-Ezra et al., 2023). Many aspects of FA pathology have been recapitulated in mice using Doxy-inducible shRNA knockdown of *Fxn* in the FXNKD mouse model (Chandran et al., 2017). The TG^+^ mouse shows progressive decline in behavioral outcomes and cardiomyopathy, but requires more analysis to determine the full nature of ataxia progression and therapeutic potential.

The FXNKD model was originally validated using a water/I.P. combination delivery of Doxy to knock down frataxin (Chandran et al., 2017), but this study confirms the ability of *ad libitum* chow-based delivery of Doxy for effective knockdown of frataxin. Overall, a similar profile of decline and variability were seen in rotarod and open field testing, but grip strength outcomes were not replicable.

In recent years, one potential therapeutic has been approved in FA, the NRF2 inducer OMAV (SKYCLARYS^®^). In clinical trials, OMAV showed a modest, but significant, effect on modified FARS (mFARS) scoring outcomes (Lynch et al., 2023). Classical FARS and mFARS testing both include facets of endurance, balance, coordination and stability, which can also be studied in mouse models using a range of tests. The NRF2 pathway is known to be downregulated in FA, and upregulation is associated with improved antioxidant response and decreased ROS (Sanz-Alcázar et al., 2024). In our study, we sought to define all phenotypic aspects of FA dysfunction and assess the effects of OMAV and DMF, both NRF2-inducing drugs, in the FXNKD mouse model to assess the level of therapeutic benefit and the potential therapeutic roadblocks of the shRNA induction model.

### Correlating mouse function with human outcomes

Mouse function can be tested in a variety of ways, including tests of muscular endurance, strength and sensation, neurologically stimulated and unstimulated. In an attempt to mirror the types of measures looked at in clinic, such as the FARS, we grouped testing types in a similar way: tests of bulbar function (not applicable and not included in mFARS testing), upper- and lower-limb coordination, upright stability and gait, and peripheral nervous system function (FARS and mFARS, Friedreich's Ataxia Research Alliance).

Upper- and lower-limb coordination were assessed by rotarod, grip strength, balance beam and Locotronic foot misplacement tests. The rotarod test revealed a significant, linear decline in endurance in the TG**^+^** mice compared to the corresponding controls (WT^−^, WT^+^ and TG^−^ mice), while the balance beam and Locotronic tests showed an increase in time needed to complete the task as ataxia worsened. However, a plateau in scoring outcome was observed, which is shown in other models of FA, such as the YG8R and YG22R mouse (Anjomani Virmouni et al., 2014). Because the balance beam and Locotronic apparatus are a fixed distance, a score over 10-15 s shows complete stoppages during testing from the animal, and these tests should therefore only be used to assess earlier disease onset. Grip strength showed no significant outcomes when normalized to body weight, and only measurement of all four paws showed a significant difference when unnormalized. The lack of reproducibility in this test suggests that it should not be included in testing of this model.

Gait analysis, open field and unstimulated rearing were also assessed as markers of upright stability/gait and coordination, similar to the stance and gait markers outlined by the FARS. Gait analysis confirmed the presence of ataxia, in which mice begin to ‘duck walk’, spreading the back paws further apart and decreasing the distance traveled with each step. By making these changes in gait pattern, mice with neurological and muscular dysfunction were able to improve their stability and walk more effectively than with their paws under the abdomen. Open field test showed a slowing in gait and a decrease in willingness to ambulate by TG**^+^** mice. An interesting point of significance was the TG**^−^** group, which also showed significantly decreased ambulation compared to the WT**^−^** and WT**^+^** groups despite it still being significantly greater than that of TG**^+^** mice.

Rearing test showed a decrease in willingness to rear (number of events) as well as a trend in the time needed to initiate a rear. During analysis, TG**^+^** mice tended to stand further back from the wall than WT**^+^** or WT**^−^** animals, which should be studied in the future using a camera below the apparatus or a mirror with an angled camera in order to measure the distance animals stand from the edge to maintain upright stability. Other models of FA and other cerebellar ataxias could also show this change in stance when rearing and should be explored. Future studies should also study this effect at multiple timepoints, as dysfunctional stability could be occurring earlier than 12 weeks of induction, and timepoints later than 12 weeks of induction will likely show an even more significant decline in rearing events and changes in stance to rear.

Although classical signs of peripheral neuropathy are not all present in human patients with FA, examination by a light touch or pinprick show a severe degree of sensory loss (Morral et al., 2010). The main clinical assessment of motor function loss in FA includes observational, low-impact analysis by a clinician using one of many ataxia scales, all of which include a sensory function aspect (Bürk et al., 2013). The correlating tests of sensory function in mice often include tests such as the hot plate test, which is not preferred for repeated measures, and Von Frey filament test to try to mimic the clinical loss of sensation to touch (de Oliviera et al., 2020). The Von Frey filament test provided an expected outcome, in which TG**^+^** mice required a larger amount of force to respond than for the corresponding controls (WT^−^, WT^+^ and TG^−^ mice). In addition to the Von Frey filament test, we were able to identify the adhesive test as another measure of peripheral sensation and function. The adhesive test showed an increase in time needed to remove the stimulus and is likely the result of an increased time to both sense the adhesive and locate it using the paws.

### Robust observational analysis of FXNKD mice

While completing the battery of tests, we also carried out observational, low-impact analyses of the mice at 8 and 12 weeks of Doxy induction. Using the ‘four-part method’, as previously described (Guyenet et al., 2010), did not allow for differentiation of TG**^+^** mice until 12 weeks of Doxy, while our more novel method, the SMAS, allowed for significant identification of phenotype at 8 weeks of induction.

The SMAS also decreased overall variability at both given timepoints, accounting for the basic measures of muscular decline (hindlimb grasping, kyphosis and gait/ledge test) and measures of what appears to be ‘accelerated aging’ in this model of ataxia. Other models of FA, such as the YG8-800, also show signs of accelerated aging, such as altered coat condition, ocular discharge/sinking and decreased body condition (Gérard et al., 2023). By including these markers, our SMAS method allows for a more robust analysis of phenotype with very little handling time or technology needed to complete. Additionally, the increased sensitivity to detect ataxias at an earlier timepoint is of value and will allow for earlier detection of disease in mouse models of FA.

The discovery of a more robust, less variable scoring method in the FXNKD mouse model will serve as a valuable tool for monitoring phenotype progression as well as assessing new therapeutics in FA mice. This testing model is likely to capture phenotypes in other cerebellum-affected ataxias in mice and should be assessed in other FA models and models of other diseases, such as amyotrophic lateral sclerosis or Leigh syndrome, especially as an addition to any therapeutic assessment in these models. By including an observational testing modality, this study better mirrored the human clinical assessment models currently in use.

### Assessment of NRF2-inducing therapeutics

While we were identifying the aspects of the TG**^+^** phenotype that best aligned with human outcomes and testing, we also tested the therapeutic use of this mouse model using two NRF2-inducing agents. OMAV was chosen as it is the current standard of care in FA, and DMF was chosen as another known potent NRF2 inducer. Induction of NRF2 was assessed by evaluating mRNA expression of the downstream target *Nqo1* in quadricep muscle after 12 weeks of Doxy. Males showed the expected FA-related decline in *Nqo1*, which was ameliorated with therapeutics, whereas females showed non-significant downregulation of *Nqo1* and no therapeutic effect. Dosed animals were also run through selected tests: rotarod, open field, Von Frey and balance beam. These tests covered coordination, gait, endurance and peripheral sensation. Of all the tests completed, no test had a drug-dependent rescue of the ataxic phenotype. Because of the marked difference in NRF2 downstream signaling between TG^+^ males and females, these data were separated by sex for analysis of therapeutic effect.

Despite significant rescue of NRF2 downstream signaling by OMAV and DMF (shown by *Nqo1* expression returning to WT^+^ levels), TG^+^ males given either drug did not show improvement in behavioral outcomes at any timepoints studied. In fact, the only noted significance occurred in the opposite direction of our hypothesis, in which DMF appears to worsen rotarod outcomes compared to those of undosed TG**^+^** mice. Females also showed a similar deficit in the rotarod when DMF was administered.

NRF2 induction alone might not be a sufficient therapeutic mechanism in FA, requiring either a combination therapy approach (NRF2 inducer along with another therapeutic mechanism) or a movement to other therapeutic mechanisms. Additionally, decrease of ROS levels through the NRF2-signaling mechanism might not result in rescue of behavioral outcomes in FXNKD muscle.

### Sexual dimorphism in the FXNKD mouse

The discovery of a differential response to NRF2 induction led to the discovery that females trend toward appearing less affected than males at any matched timepoint, which has been shown at 18 weeks of Doxy induction (Perry et al., 2024) but is only trending in our data at 12 weeks of Doxy induction. Sexual dimorphism has been noted in other mouse models of FA (Fil et al., 2023; Salinas et al., 2024). The survival effects have been studied to an extent with nicotinamide treatments, but survivor bias potentially complicates the data (Perry et al., 2024). The time course of sexual dimorphism should be better studied using the robust methods of phenotyping we have identified in this study and would likely elucidate a point when the female phenotype ‘catches up’ in severity to that of males. Survival outcomes show that male mortality seems to occur over time, whereas female mortality occurs in a smaller window of time. This also requires further study but could suggest that the male and female mice model two different forms of FA, early- and late-onset, or with different cardiac modeling over time, resulting in death. Sex-based hormonal differences could also play a role in phenotypic timeline.

### Other takeaways and limitations of the study

As seen in a few different phenotypic markers (rotarod, open field, rearing and adhesive test), we concluded that the TG**^−^** mouse might still have residual *Fxn* knockdown and therefore exhibit a mild FA phenotype due to possible leaky expression of the shRNA transgene. This suggests that the TG**^−^** mouse could be more similar to a reduced expression mouse, not a WT**^+^** or WT**^−^** littermate. For future studies, WT**^+^** littermates should be prioritized as controls to prevent a partial phenotype in TG**^−^** mice from affecting the dynamic range in behavioral experiments, as well as in mRNA levels, while still controlling for the presence of Doxy. However, the TG**^−^** mouse could be important to include in studies of metabolic or proteomic profiles.

Other limitations of the study include the manual Von Frey apparatus, which is not as continuous in measurements as a newer electronic apparatus, and restrictions on the number of tests that could be completed in week 12, resulting in smaller *n* number for some assays studied. Another limitation is the focus on NRF2-inducing therapeutics, which are one small class of potential therapeutics for FA. Other classes of therapeutics or combination therapies with NRF2-inducing therapeutics could increase the therapeutic benefit in the FXNKD mouse model or other mouse models of FA.

## Conclusions

The FXNKD Doxy-inducible (TG**^+^**) mouse exhibits a progressive ataxic decline mirroring that of the human patient. By subjecting the TG**^+^** mouse to tests of coordination, gait, stability and peripheral sensation, we were able to mirror the phenotypic decline seen and measured in humans by tests such as the FARS over a 12-week period of Doxy induction. The progression of this model could be one of the most clinically relevant for this purpose, as many models of FA do not develop severe ataxia or early-onset death from cardiomyopathy as seen in the FXNKD (Chandran et al., 2017).

However, this model shows a sexual dimorphism that complicates therapeutic testing. Additionally, therapeutic testing with NRF2-inducing molecules failed to provide a behavioral effect and did not show the expected target engagement in females. Downstream target *Nqo1* was not significantly altered by Doxy induction in females and was not affected by OMAV or DMF treatment. Although males showed the expected downstream signaling changes in the TG**^+^** mice and the OMAV/DMF-dosed mice, behavioral outcomes did not change as a result of NRF2 induction.

These data suggest that the FXNKD mouse shows a very strong phenotype that should be investigated further to gain more knowledge on the FA condition. This model could be useful in testing of other therapeutics, but baseline NRF2 signaling was not uniformly affected between FA males and females, and NRF2 induction did not cause the hypothesized benefit on neurobehavioral and skeletomuscular outcomes.

## MATERIALS AND METHODS

### Animal development

All animal protocols were approved by the Institutional Animal Care and Use Committee at the University of California, Davis and were also in accordance with the National Institutes of Health (NIH) guidelines for the Care and Use of Laboratory Animals. Male and female FXNKD mice were generated as previously described on a C57BL/6J background (Chandran et al., 2017). Heterozygous FXNKD mice were bred in-house with WT littermates or C57BL/6J mice (every other generation) to prevent genetic drift. Mice were housed individually in polycarbonate cages during the study, to ensure adequate daily food intake visually as well as better monitoring of health. Body weights were monitored weekly. Mice were provided with Mouse Igloos (Bio-Serv) to provide additional shelter and housed within view of multiple other cages of animals. Total group sizes ranged from *n*=4 to *n*=45 and included approximately half males and half females. Male and female data are reported combined in Figs 1-4, and, after discovering a sexual dimorphism in therapeutic response, they are reported separately in Figs 5 and 6 (overlapping control groups in all figures). Sample sizes are listed in figure legends and vary owing to molecular and behavioral tests requiring different power, removal or replacement of some tests (such as Locotronic after noting high variability), or lack of time in week 12 to complete all tests in all mice. See ‘Behavior testing’ section for further breakdown.

### Genotype analysis

We prepared genomic DNA from ear clip samples collected from the mice by using a standard HotShot DNA extraction method, and PCR-based genotyping was completed using published primer sets (Chandran et al., 2017) for WT and shRNA (Integrated DNA Technologies). Animals with one copy of the transgene were identified as transgenic mice (‘TG’) and the littermates with no shRNA transgene present were identified as ‘WT’ littermates (Table 1).

### Doxy administration

Mice were group housed and provided food and standard chow (Teklad 2018, Inotivco) *ad libitum* from weaning until they reached 12-15 weeks of age. At that age, animals either remained on control chow or were given a Doxy chow (200 ppm, Teklad 2018 base, Inotivco) to induce loss of FXN. Mice for biochemical testing and behavioral testing were administered Doxy chow for 12 weeks before endpoint, while survival animals had behavior measured through 12 weeks of Doxy and allowed to stay on treatment until humane endpoint or death occurred. Animals were assigned to diet groups by counter-balancing body weights before Doxy induction. Animals were fed *ad libitum* but given a limited number of pellets at feeding to visually track daily food consumption. Animals of either genotype that were given a control chow are denoted with a ‘−’ (WT**^−^** or TG**^−^**) and animals given Doxy chow are denoted with a ‘+’ (WT**^+^** or TG**^+^**), as described in Table 1.

### Peanut butter dosing

At the beginning of the fourth week of Doxy feeding, all animals studied were assigned to daily treatment with 0.2 ml plain (placebo) peanut butter or peanut butter containing a NRF2-inducing drug, OMAV (24 mg kg^−1^) or DMF (82 mg kg^−1^). Dosing was performed and continued daily in afternoons through 12 weeks of Doxy induction.

### Tissue collection

After completing the week 12 behavior tests, animals were euthanized with pentobarbital overdose followed by exsanguination under deep anesthesia. The cerebellum and quadricep muscle were removed and washed with phosphate buffered saline before flash freezing in liquid nitrogen. Tissues were pulverized with a frozen mortar and pestle held on dry ice, then aliquoted for RNA isolation.

### RNA isolation and quantitative real-time PCR

RNA was extracted from quadricep muscle and cerebellum aliquots using an RNeasy Fibrous Tissue Kit (Qiagen) and RNeasy Lipid Tissue Kit (Qiagen) according to the kit instructions. Complementary DNA (cDNA) was synthesized using an iScript cDNA Synthesis Kit (Bio-Rad). Quantitative real-time PCR was carried out using PowerUp SYBR Green Master Mix (Thermo Fisher Scientific) on a QuantStudio 7 Pro Real-Time PCR System (Thermo Fisher Scientific). Primer sequences can be found in Table S4*.* The data were analyzed using delta-delta CT calculation with the WT**^−^** group used as the control group. Ribosomal protein 18S (*Rps18*) was used as a reference gene for the quadricep; beta-actin (*Actb*) was used as a reference gene for the cerebellum.

### Behavior testing

Male and female mice underwent behavior testing starting at Doxy chow initiation and continuing for 12 weeks of *ad libitum* feeding. Behavior tests included the following: rotarod, grip strength, adhesive, Von Frey filament, rearing, manual gait analysis, open field, balance beam, Locotronic foot misplacement and ataxia scoring. All tests are described in detail below. In all weeks besides week 12, only one test was completed per day. During week 12, up to two total tests were completed per day with at least 3 h in between tests. Animals were provided food and water directly before and after all tests. Rotarod, balance beam, grip strength and Locotronic tests were the only tests that were undertaken weekly. Each animal underwent rotarod and balance beam testing, while one subset completed grip strength testing and another subset completed Locotronic testing, hence a smaller sample size for those two assays. Adhesive and rearing testing were also unable to be performed on all mice during week 12, leading to smaller sample sizes. Experimenters were made unaware of treatments during testing by covering treatment cards. For any video-based analysis (rearing, adhesive tests), only animal ID and date were written on a whiteboard, and videos were analyzed in random order. For gait analysis, subsections of the paper walked on were cut and scanned and assigned random IDs for analysis. For software-based tests (open field, Locotronic), the software automatically generated distances and times.

#### Grip strength

The grip strength test was run using a digital push-pull meter (Imada) as previously described (Roberts et al., 2018) with either a single metal bar or a wire grid (∼4 inches in diameter with 1 cm grid spacing). To test all four limbs, three trials were completed using the wire grid. To test front limbs only, three trials were completed the subsequent day to prevent fatigue. As in the original paper (Chandran et al., 2017), the maximum normalized grip strength per day was reported (***g*** force/g body weight). Raw values were also compared in Fig. S1.

#### Open field

The open field test was conducted in a 40×40×40 cm white acrylic box. Mice were placed in one corner of the box and allowed to freely explore the arena for 15 min. Videos were recorded by a camera mounted on the ceiling directly above the apparatus. The videos of mouse movement were tracked using Ethovision XT17 software (Noldus). The center zone was set as a 25×25 cm area in the middle of the arena. Total distance traveled and total time spent in the center zone were automatically computed using the software.

#### Rotarod

Motor coordination ability was assessed weekly using an Ugo-Basile accelerating rotarod treadmill. Three trials were performed, with the speed of the rotation gradually increasing in a linear manner from 4 to 40 rpm, and each trial lasting up to a maximum time of 5 min, separated by a rest period of 10-20 min between each trial. The time taken for the mouse to fall from the apparatus was recorded, and the maximal value for each week was reported.

#### Adhesive test

The adhesive removal test was performed as previously described (Schallert et al., 1982) using removable 0.25 inch colored stickers (Avery). The mouse was lightly restrained and had a sticker placed securely on the snout, ∼1 mm above the nostrils/whiskers, before being placed in a clean cage with the bedding removed. The mouse was video recorded, and latency to initiate a stimulus-directed response as well as latency to remove the stimulus from the body were quantified.

#### Von Frey test

Tactile stimulation of the hind paws was presented by means of a variety of manual Von Frey filaments. Once animals had acclimated to the Von Frey grid for >15 min in the home room, a 0.4 g filament was first utilized on their back paw, and the up-down method was used to determine subsequent fiber sizes as previously described, allowing five subsequent measurements after the first stimulus-directed response (McMackin et al., 2016). Stimulus-directed response is defined as an immediate reaction to the fiber and can include withdrawal of paw, immediate grooming of paw, head turn toward stimulus or body turn toward stimulus. The lowest force required to illicit a response was reported. The same researcher completed all testing while unaware of animal identification to prevent researcher-to-researcher bias.

#### Gait analysis

Gait analysis was performed at endpoint by allowing the animals to walk through a 50 cm-long, 10 cm-wide runway that was lined with thin white exam paper. Fore and hind paws were coated with nontoxic red and blue paint, respectively, and the mouse was allowed to walk across the exam paper. Footprints were captured on the exam paper and allowed to dry before analyzing. If steps were too smudged or the animal did not walk in a straight line, the animal had its paws repainted and was allowed to walk again. The papers were scanned, and the images were measured using ImageJ for calculating the stride lengths, spread and other parameters, as previously described (Carter et al., 2001; Wertman et al., 2019).

#### Balance beam

Balance performance was measured by placing mice on a 100 cm-long level wooden beam elevated ∼16 inches above the countertop, with a line marked 10 cm from each end as the start and end lines. All lights were turned on in the room as an aversive stimulus, and an enclosed shelter was placed at the end of the beam. A training beam of 21 mm diameter was used for two training trials, with at least 10 min of rest allowed for each animal between beam crosses. Subsequently, three trials were conducted using a 16 mm diameter beam. Time was recorded for each animal to cross from start to finish for all five trials. Additionally, a video camera recorded every trial and was later processed for quantification of foot slips as errors, as well as to confirm cross times. The experimenter who processed video to record times and errors was unaware of treatment groups. If animal showed unwillingness to move, the experimenter would ‘encourage’ movement by lightly pinching around the base of the spine.

#### Rearing test

The apparatus consisted of plexiglass walls, with two 10 cm-long opaque walls on sides and a 20 cm opaque back. The front of the apparatus was clear to enable video recording, and a mirror was used as the floor. Mice were placed into the apparatus and allowed to explore for 1 min and 30 s. Videos were recorded and scored after the test. The number of total rears was recorded as well as the time to first rear. A ‘rear’ was defined as the mouse standing up and putting one or both forepaws on the side of the cylinder, and separate rearing behaviors required the animal to place both paws back on the floor between actions.

#### Locotronic foot misplacement

Mouse limb coordination was tested once weekly using Bioseb Locotronic Foot Misplacement Apparatus, which comprised a horizontal ladder with a 5 cm-wide corridor leading to a dark arrival box, with the home cage directly outside the opening. Mice were given one training run before data collection and then underwent one trial with no rungs removed and one trial with three rungs removed (‘traps’). Time to move from the starting rung to the arrival box was automatically calculated by infrared sensors. Because mice tended to stick their head between the rungs in the traps trial, the software output for scoring foot slips was confounded and therefore not analyzed.

#### Ataxia scoring

Four-part cerebellar ataxia scoring was done as previously described (Guyenet et al., 2010). For the newly developed scale, referred to as the SMAS, the authors sought to define a more sensitive ataxia scale in ataxic mice using the four-part test as a basis and including aspects of the frailty index (Whitehead et al., 2014). Because we noted that FXNKD mice continuously showed an ‘advanced aging’ phenotype, assimilating multiple aspects of the frailty index allowed for more measured values and therefore a decrease in variability. The full list of indices measured, as well as the scores and clinical signs associated, are in Table S3*.*

### Statistical analysis

Data were analyzed using one-way or two-way ANOVA with Tukey's multiple comparisons (unless otherwise noted) using GraphPad Prism software, Version 10 (RRID:SCR_002798), with *P*<0.05 considered statistically significant. Non-parametric data were analyzed using Kruskal–Wallis test with Dunn's multiple comparisons, with *P*<0.05 considered statistically significant. Survival analysis was assessed using the Mantel–Cox (Log-Rank) test. Outliers were identified using GraphPad Prism Grubb's outlier test with alpha *P*=0.05 within each group dataset and removed from analysis. All data are presented as mean±s.e.m. unless otherwise noted. Main effects and *F* statistics for all figures are listed in Table S2.

## Supplementary Material

10.1242/dmm.052128_sup1 Supplementary information
