# Supplementary material for: Robust behavioral assessment of the inducible Friedreich's ataxia mouse does not show improvement with NRF2 induction
Source: Dis Model Mech. 2025 Apr 2;18(3):dmm052128. doi: 10.1242/dmm.052128 (PMC11992351; doi:10.1242/dmm.052128)
Supplement: Supplementary information [file dmm-18-052128-s1.pdf]

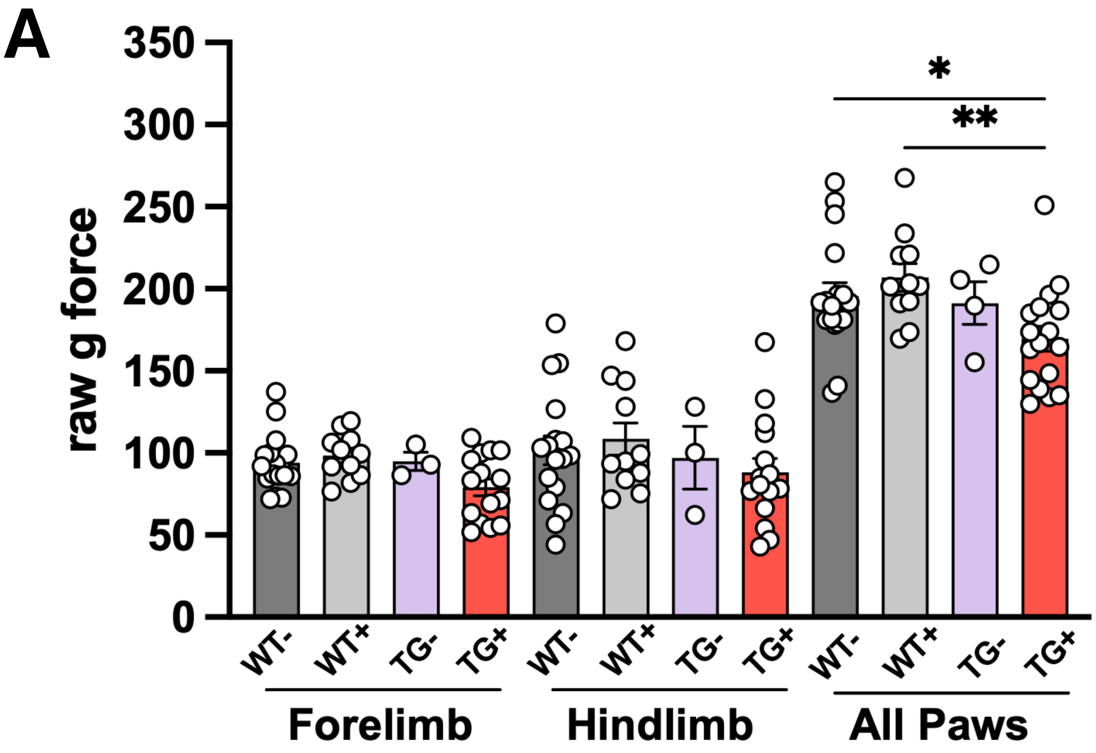

B

	Un-normalized Outcomes (gForce)		
**Genotype/Diet:**	**Forelimbs only**	**Hindlimbs (All limbs minus Forelimbs)**	**All limbs/paws**
**WT-**	93.9 ± 4.2 (17)	101.6 ± 8.7 (17)	195.5 ± 8.4 (17)
**WT+**	98.3 ± 1.3 (11)	108.6 ± 3.0 (11)	206.9 ± 2.5 (11)
**TG-**	94.8 ± 5.5 (3)	97 ± 19.1 (3)	191.3 ± 13.1 (4)
**TG+**	79.1 ± 5.1 (15)	88.1 ± 8.6 (15)	169.7 ± 7.5 (17)
p-value (vs. WT+)	p=0.3366	p=0.2777	** *p=0.0057*

Fig. S1. Un-normalized grip strength. (A/B) Un-normalized grip strength outcomes do not show significance for front limbs or hindlimbs (four minus front values) only, but did show a significant difference in the testing of grip strength in all four limbs (n = 3-17). **A**: 2-way ANOVA, **B**: mean ± s.e.m. (n)

Disease Models & Mechanisms • Supplementary information

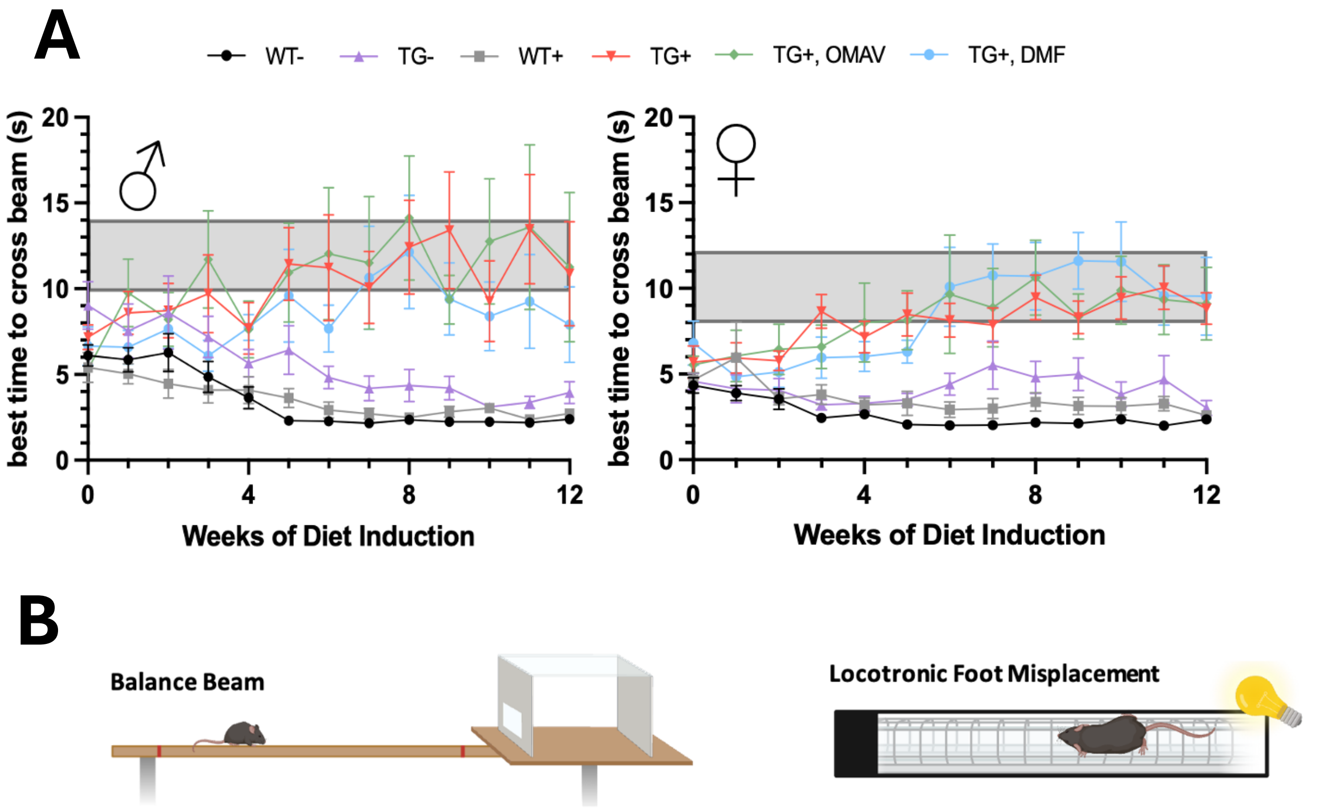

Fig. S2. Balance beam testing over time shows a trend toward improved female outcomes. (A) Balance beam maximal score plateaus by week 5-6 at approximately 10-14 seconds for males to cross the designated zone (shown by the transparent grey bar), and females approximately 9-12 seconds, in which a longer time would indicate a complete stoppage of movement from the mouse (and therefore confound the maximal time to cross). Females show an overall lesser "maximum" value than males, with less variability (*n* = 8-14). **(B)** Balance beam time to cross was measured using a stopwatch, started when mouse shoulders crossed the "start" line and ended when shoulders crossed the "finish" line. A wooden box was placed at the end of the beam as a positive stimulus, where mice could rest for 15-20 seconds after each trial. Locotronic apparatus had a built-in light, and animals crossed ladder rungs from light side to dark side. Home cage was placed directly outside as a positive stimulus. Created in BioRender by Montgomery, C. B. (2024). https://BioRender.com/h41j819. This figure was sublicensed under CC-BY 4.0 terms.

Disease Models & Mechanisms • Supplementary information

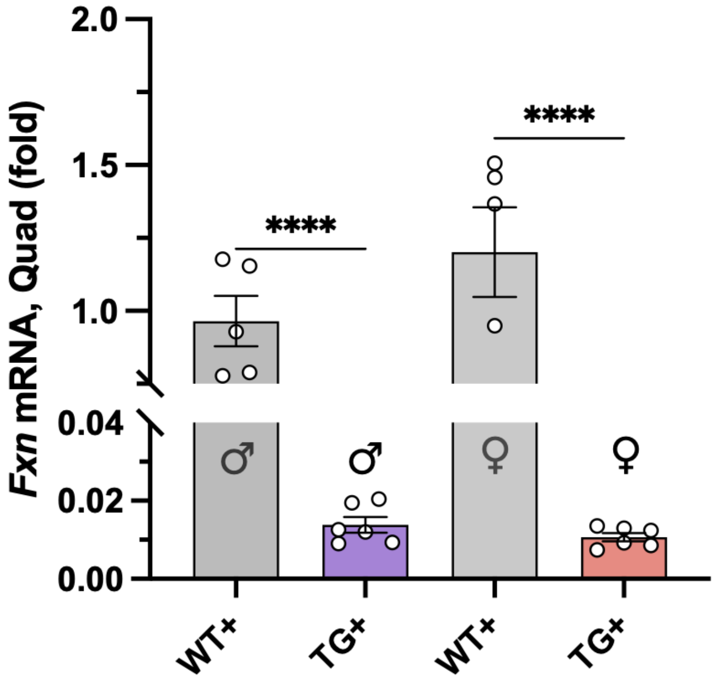

Fig. S3. *FXN* mRNA levels in quadricep. Quadricep (Quad) shows more >98% decrease in *Fxn* mRNA levels in both male (purple) and female (red) FXNKD mice.

Disease Models & Mechanisms • Supplementary information

Table S1. Normalized grip strength outcomes

Grip strength was measured after 12 weeks of Doxy induction, with front and four limb tests completed on consecutive days. All three analyses showed insignificant outcomes. Reported as mean ± s.e.m. (*n*)

	Normalized Outcomes (gForce/gBody Weight)		
**Genotype/Diet:**	**Forelimbs only**	**Hindlimbs (All limbs minus Forelimbs)**	**All limbs/paws**
**WT-**	2.1267 ± 0.108 (17)	4.0589 ± 0.330 (17)	7.888 ± 0.378 (17)
**WT+**	2.1479 ± 0.041 (11)	4.0425 ± 0.124 (11)	7.679 ± 0.136 (11)
**TG-**	2.0367 ± 0.238 (3)	4.2010 ± 1.097 (3)	8.405 ± 0.997 (4)
**TG+**	2.2120 ± 0.145 (15)	3.5527 ± 0.303 (15)	6.995 ± 0.328 (17)
p-value (TG+ vs. WT+)	p=0.9982	p=0.4347	p=0.5551

Table S2. Table of statistical method and main effects/F statistics for all figures.

Abbreviations: B: bottom, KWstat: Kruskal-Wallis statistic, MC: multiple comparisons test, ME: mixed effects, T: top

	**Analysis Type**	**Post-Hoc**	**F statistic**	***p* value**	**Sig.**
**Figure 1B**	2-way ANOVA	Tukey's MC	F (3,60)=10.75	< 0.0001	****
**Figure 1D**	2-way ANOVA	Tukey's MC	F (36,1419) = 3.332	< 0.0001	****
**Figure 1E**	ME 2-way ANOVA	Tukey's MC	F (84, 1086) = 5.831	< 0.0001	****
**Figure 1F**	2-way ANOVA	Tukey's MC	F (3, 83) = 0.5624	0.6413	n.s.
**Figure 2CT**	1-way ANOVA	Tukey's MC	F (3, 35) = 23.18	< 0.0001	****
**Figure 2CB**	1-way ANOVA	Tukey's MC	F (3, 33) = 19.49	< 0.0001	****
**Figure 2E**	1-way ANOVA	Tukey's MC	F (5, 87) = 33.46	< 0.0001	****
**Figure 2F**	1-way ANOVA	Tukey's MC	F (5, 109) = 14.26	< 0.0001	****
**Figure 2G**	2-way ANOVA	Tukey's MC	F (3, 46) = 0.6048	0.6152	n.s.
**Figure 3C**	1-way ANOVA	Tukey's MC	F (3, 26) = 8.467	0.0004	***
**Figure 3E**	1-way ANOVA	Tukey's MC	F (3, 30) = 1.702	0.1877	n.s.
**Figure 3G**	1-way ANOVA	Tukey's MC	F (3, 78) = 14.12	< 0.0001	****
**Figure 3I**	1-way ANOVA	Tukey's MC	F (3, 25) = 4.108	0.0169	*
**Figure 4B-8w**	Kruskal-Wallis	Dunns' MC	KWstat = 0.8125	0.8315	n.s
**Figure 4B-12w**	Kruskal-Wallis	Dunns' MC	KWstat = 48.79	< 0.0001	****
**Figure 4D-8w**	Kruskal-Wallis	Dunns' MC	KWstat = 11.34	0.0100	*
**Figure 4D-12w**	Kruskal-Wallis	Dunns' MC	KWstat = 55.97	< 0.0001	****
**Figure 5C**	2-way ANOVA	Tukey's MC	F (3, 33) = 2.774	0.0568	n.s.
**Figure 5D**	ME 2-way ANOVA	Tukey's MC	F (36, 618) = 3.706	< 0.0001	****
**Figure 5E**	ME 2-way ANOVA	Tukey's MC	F (36, 631) = 3.695	< 0.0001	****
**Figure 5F**	2-way ANOVA	Tukey's MC	F (3, 65) = 1.184	0.3226	n.s.
**Figure 6A**	2-way ANOVA	Tukey's MC	F (3, 84) = 0.4465	0.7204	n.s.
**Figure 6B**	2-way ANOVA	Tukey's MC	F (3, 54) = 1.280	0.2906	n.s.
**Figure 6C-8w**	Kruskal-Wallis	Dunns' MC	KWstat = 7.643	0.0219	*
**Figure 6C-12w**	Kruskal-Wallis	Dunns' MC	KWstat = 18.08	0.0001	***
**Figure 6D-8w**	Kruskal-Wallis	Dunns' MC	KWstat = 2.000	0.3679	n.s.
**Figure 6D-12w**	Kruskal-Wallis	Dunns' MC	KWstat = 14.64	0.0007	***
**Figure 6E**	2-way ANOVA	Tukey's MC	F (1, 16) = 0.004548	0.9471	n.s.
**Figure 6F**	Mantel-Cox	N/A	χ^2 = 11.76, df = 2	0.0028	**

Disease Models & Mechanisms • Supplementary information

Table S3. Full description of SMAS scoring parameters. Grey cells overlap with the 4-part method

Salinas-Montgomery Ataxia Scale (SMAS) Full Description		
**Test**	**Score**	**Description of Scoring**
**Ledge test**	0	Minor 1-2 slips with controlled walking pattern
	0.25	1-2 minor slips, less controlled walking, but controlled descent
	0.5	Multiple slips, may take breaks to catch, uses tail to balance, or lands unbalanced
	1	Falls off ledge, refuses to move, lands on head
**Hindlimb grasping**	0	Normal (legs spread away from abdomen, toes kept straight)
	0.25	Toes/paw curled for at least 50% of time without full retraction of limb
	0.5	One limb fully retracted/curled for at least 50% of time
	1	Both limbs retracted/curled at least 50% of time
**Gait Disorders**	0	No abnormality
	0.25	Minor spreading, slightly abnormal step path
	0.5	Notable spreading, abnormal step path
	1	Significant spreading, dragging of abdomen
**Kyphosis**	0	Curvature absent
	0.25	Mild curvature only when standing still
	0.5	Mild curvature present at all times
	1	Clear evidence of hunched posture, maintains persistent kyphosis
**Alopecia/Coat Condition**	0	Smooth, sleek, shiny fur with normal hair density
*only on singly housed mice, to avoid barbering	0.5	Coat is slightly ruffled, thinning
	1	Unkept, ungroomed, extensive fur loss (>25% or patches over 0.5cm^2)
**Eye Discharge/Sinking**	0	Normal size, no secretions
*do not measure in mice with congenital microphthalmia	0.5	Slight swelling around eyes and/or secretions, one or both eyes slightly shrunken
	1	One/both eyes <50% of expected size, severe secretions, may cause to crust over
**Body Condition Score**	0	BCS > 2.5
(BCS)	0.5	BCS 2-2.5
	1	BCS <2
**Tail Stiffening**	0	No stiffening, quickly curls around finger
	0.5	Tail responds to stimulus but cannot curl around finger
	1	Tail is completely unresponsive
**Righting Reflex**	0	Normal landing, on all four limbs
	0.5	Animal lands on side or back but rights itself immediately
	1	Animal lands on back and is not able to immediately right itself
**Tremor**	0	No tremor
	0.5	Slight tremor, animal can still ambulate/rear
	1	Marked tremor, animal cannot rear

Table S4. Primer sequences used in qRTPCR

Gene Name	Forward (5'->3')	Reverse (5'->3')
Fxn	GATCAACAAGCAGACCCCAAA	AGGCCAATGAAGACAAGTCCA
Nqo1	ACAGGTGAGCTGAAGGACTC	CCAAACCACTGCAATGGGAA
Rps18	ATGCAGAACCCACGACAGTA	TTCTTCAGCCTCTCCAGGTC
Actb	TTACTGCTCTGGCTCCTAGC	CCTGCTTGCTGATCCACATC

Disease Models & Mechanisms • Supplementary information
